# Effects of cognitive stimulation on neuropsychiatric symptoms in elderly with Alzheimer's disease: A systematic review

**DOI:** 10.1590/S1980-5764-2016DN1003003

**Published:** 2016

**Authors:** Raiana Lídice Mór Fukushima, Elisangela Gisele do Carmo, Renata do Valle Pedroso, Pollyanna Natalia Micali, Paula Secomandi Donadelli, Gilson Fuzaro, Reisa Cristiane de Paula Venancio, Juliana Viola, José Luiz Riani Costa

**Affiliations:** 1 Mestrando. Universidade Estadual Paulista "Júlio Mesquita Filho", Departamento de Educação Física, Instituto de Biociências, Rio Claro SP, Brazil; 2 Mestre. Universidade Estadual Paulista "Júlio Mesquita Filho", Departamento de Educação Física, Instituto de Biociências, Rio Claro SP, Brazil; 3 Doutorando. Universidade Estadual Paulista "Júlio Mesquita Filho", Departamento de Educação Física, Instituto de Biociências, Rio Claro SP, Brazil; 4 Doutor. Universidade Estadual Paulista "Júlio Mesquita Filho", Departamento de Educação Física, Instituto de Biociências, Campus Rio Claro SP, Brasil

**Keywords:** Alzheimer's disease, depression, apathy, anxiety, dementia, cognitive stimulation

## Abstract

**Introduction:**

Neuropsychiatric symptoms are frequent in Alzheimer's disease and negatively affect patient quality of life.

**Objective:**

To assess the effectiveness of cognitive stimulation on neuropsychiatric symptoms in elderly patients with Alzheimer's disease.

**Methods:**

The included articles were reviewed between December 2015 and June 2016, and the inclusion criteria were: (1) studies involving older adults diagnosed with Alzheimer's disease; (2) studies published in English, Spanish or Portuguese; (3) studies that determined the effect of cognitive stimulation on neuropsychiatric symptoms in elderly patients with Alzheimer's disease; (4) controlled trials.

**Results:**

Out of the total 722, 9 articles matched the inclusion criteria. Depression, apathy and anxiety were the most frequent symptoms.

**Conclusion:**

Studies reported significant results post-treatment, suggesting cognitive stimulation can be effective for these neuropsychiatric symptoms, thus improving the quality of life of Alzheimer's disease patients and their caregivers.

## RESUMO

**Introdução:**

Sintomas neuropsiquiátricos são frequentes na doença de Alzheimer e afetam negativamente a qualidade de vida destes pacientes.

**Objetivo:**

Avaliação da eficácia da estimulação cognitiva sobre os sintomas neuropsiquiátricos em idosos com doença de Alzheimer.

**Métodos:**

Os artigos incluídos foram revisados no período de Dezembro (2015) à Junho (2016) e os critérios de inclusão foram: (1) estudos com idosos com diagnóstico de doença de Alzheimer; (2) estudos publicados em Inglês, Espanhol e Português; (3) estudos que verificaram o efeito da estimulação cognitiva nos sintomas neuropsiquiátricos em idosos com doença de Alzheimer; (4) ensaios controlados.

**Resultados:**

Do total de 722, 9 artigos corresponderam aos critérios de inclusão. Depressão, apatia e ansiedade foram os distúrbios mais frequentes.

**Conclusão:**

Após tratamento, estudos relataram resultados significativos, o que sugere que a estimulação cognitiva pode ser eficaz nestes sintomas neuropsiquiátricos, melhorando assim, a qualidade de vida de pacientes com doença de Alzheimer, bem como, de seus cuidadores.

## INTRODUCTION

The aging population is a present reality in developing and developed countries worldwide. According to the World Health Organization (WHO), between 2015 and 2025, the proportion of people aged 60 years and over is set to almost double from 12% to 22%.^1^ However, a possible consequence of a high number of long-lived people is an increased prevalence of chronic diseases such as dementia.

Approximately 46.8 million individuals were diagnosed with dementia and prevalence rates are predicted to reach a startling 131.5 million worldwide by 2050.^2^ One of the most common types of dementia is Alzheimer's disease (AD). The International Classification of Disease, 10^th^ revision (ICD-10), describes AD as a neurodegenerative process^3^ characterized by progressive memory loss and other cognitive changes. According to one investigation, AD structural brain changes (i.e. cerebral atrophy) may have important effects on functional status, however, the prominent impact is on cognition and behaviour.^4^

Once diagnosed with AD, almost all people develop neuropsychiatric symptoms (NPS) at some stage during the course of the illness.^5^ Symptoms may be observed in very early stages of the disease.^5^ Such changes include depression and apathy and are characterized by wandering, agitation, resisting caregiver support, decreased emotional or behavioral control, disorientation, confusion and communication skills.^6^

According to a systematic review and meta-analysis of 48 investigations, apathy in AD patients is the most prevalent symptom (49%), followed by depression (42%), aggression (40%), anxiety (39%) and sleep disorders (39%), which explains why these are the most addressed symptoms. Less prevalent symptoms included irritability (36%), eating disorders (34%), aberrant motor disorders (32%), delusion (31%), disinhibition (17%), and hallucination (16%). Euphoria was the least common, with 7% occurrence in AD patients.^7^ The development of NPS in AD can negatively influence and accelerate disease progression with early institutionalization, as well as interfere with treatment effects and prognosis.^8^ ^,^ ^9^

Another study conducted in 2010, which included 29 AD and 13 Vascular Dementia (VD) patients, showed that most AD patients presented significant NPS, such as depression and anxiety, and that as the illness progresses there was an increased prevalence of psychotic symptoms, such as hallucinations and delusions, which tend to be associated with paranoia.^10^

As stated in an annual AD report, pharmacologic treatment for dementia patients is expensive and expected to reach $1 trillion dollars (US) worldwide in 2018, rising to $2 trillion dollars (US) by 2030.^11^ Currently, no effective pharmacologic treatment or drug has been established to cure or reverse the deterioration caused by AD, where treatment is intended to manage the symptoms.^12^ In fact, only a few drug treatments are useful for NPS in AD patients.^5^ In this context, non-pharmacological interventions are considered a useful strategy due to their lower costs and almost complete absence of adverse effects in managing behavioral symptoms and compensating for cognitive impairments. Among various non-pharmacologic treatments, cognitive stimulation (CS) and cognitive training (CT) are prospective options for individuals with dementia.^18^ Also, CS can be considered and may have beneficial effects on AD behavioral symptoms in the elderly. CS promotes involvement in activities that are aimed at general enhancement of global cognitive and social functioning, without particular objectives.^18^ By contrast, CT usually involves guided practice of standard tasks in order to enhance or maintain specific cognitive functions (i.e. memory). However, as stated by the same authors, it can be very difficult to distinguish between stimulation and training programmes.^18^ ^,^ ^19^

Thus, all CS programs aim to optimize cognitive status within a socially-oriented context through an integrative and inclusive approach.^4^ These programs are known to impact cognitive reserve, which is generally known to delay global cognitive and functional expression of neurodegenerative diseases. Few investigations have addressed the benefits of cognitive stimulation in neuropsychiatric symptoms of AD.^21^ ^,^ ^23^ ^-^ ^29^ Therefore, the purpose of the present review was to provide further evidence of the effectiveness of cognitive stimulation in neuropsychiatric symptoms among elderly with AD. 

## METHODS

The methodological process in this study was based on a systematic literature review, guided by bibliographic searches in the following databases: *Web of Science, Scopus, PsycINFO* and *Medline/*PUBMED. These databases were chosen because they specifically approach topics associated with health. Boolean operators and the keywords utilized were: (Alzheimer dementia OR Alzheimer disease OR Alzheimer) AND (cognitive stimulation OR global stimulation OR group therapy AND (neuropsychiatric disturbances OR neuropsychiatric disorders OR neuropsychiatric symptoms OR depression OR agitation OR apathy OR insomnia) NOT transcranial). There were no restrictions concerning the publication date of the papers and all included articles were reviewed between December 2015 and June 2016. Besides the search in the databases, we also carried out a manual search in the reference lists of the selected papers. The following inclusion criteria were used: (1) studies involving elderly diagnosed with Alzheimer's disease; (2) studies published in English, Spanish or Portuguese; (3) studies that determined the effect of cognitive stimulation on neuropsychiatric symptoms for elderly with Alzheimer's disease; (4) controlled trials. Those papers not meeting these inclusion criteria were excluded from this review.

## RESULTS SUMMARY

**Figure 1:**
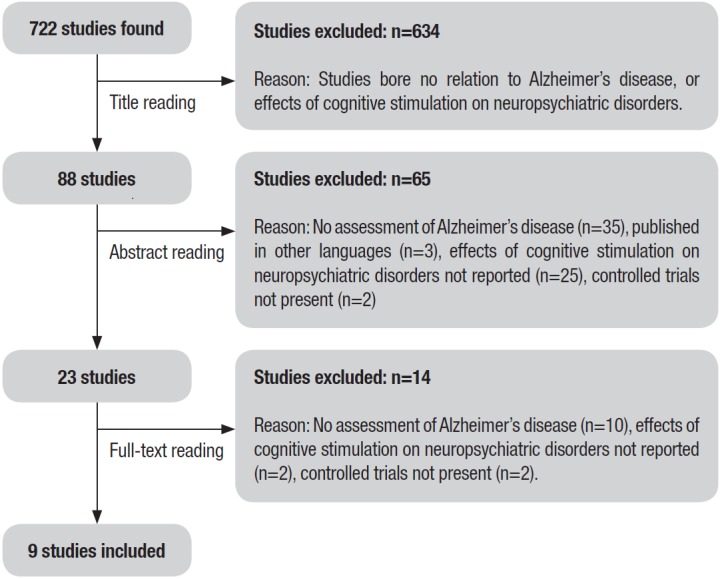
Flowchart illustrating the different phases of search and study selection

Search results. The literature search yielded a total of 722 papers. After initial screening, 634 were excluded as they bore no relation with the aim of this review. The next step was based on reading the abstracts of the remaining 88 studies, of which 62 were excluded, as they did not match any inclusion criteria. Thus, 23 studies met the criteria for full-text review, of which 14 were subsequently eliminated because they did not include samples diagnosed with Alzheimer's disease (n=10), did not verify the effect of cognitive stimulation on neuropsychiatric symptoms in elderly with Alzheimer's disease (n=2), and were not controlled trials (n=2) (Figure 1). A final total of nine papers was therefore included in this review. 

Descriptive results. Table 1 provides a detailed summary of the selected investigations. 

**Table 1:**
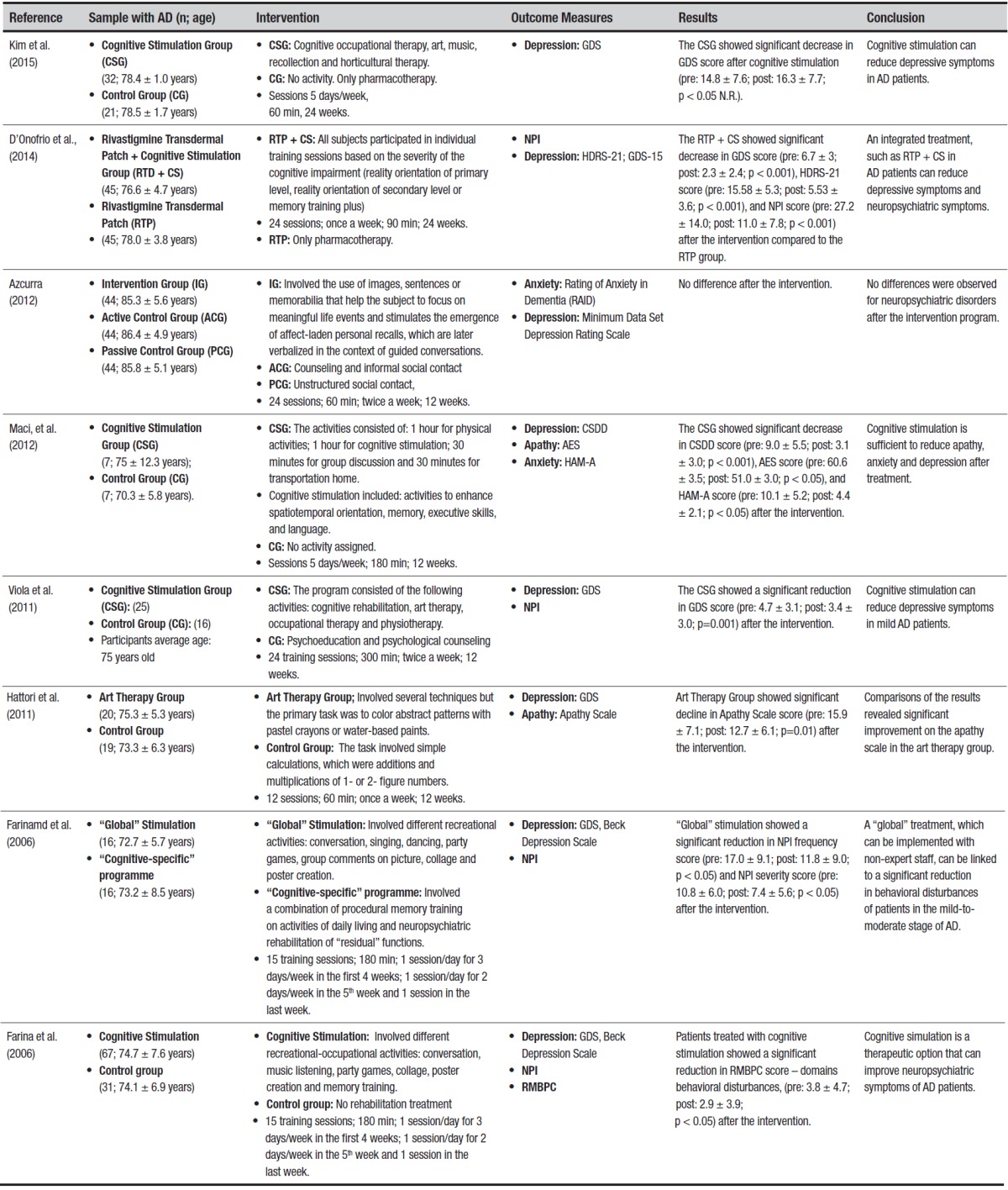
Characteristics and results of studies included in final selection.

GDS: Geriatric Depression Scale; NPI: Neuropsychiatric Inventory; HDRS-21: Hamilton Rating Scale for Depression; RAID: Rating Anxiety in Dementia; CSDD: The Cornell Scale for Depression in Dementia; AES: Apathy Evaluation Scale; HAM-A: Hamilton Anxiety Rating Scale; RMBPC: Revised Memory and Behavior Problems Checklist.

Among the studies analyzed, participants were diagnosed with Alzheimer's disease according to the Diagnostic and Statistical Manual of Mental Disorders- IV (DSM-IV),^21^ ^,^ ^22^ and by the National Institute of Neurological and Communicative Disorders and Stroke and the Alzheimer's Disease and Related Disorders Association (NINCDS-ADRDA).^23^ ^-^ ^29^ Only one study investigated for the presence of diffuse brain atrophy on Magnetic Resonance Imaging (MRI) and decreased blood flow in the parietal lobe and posterior cingulate gyrus on single emission computed tomography (SPECT).^27^

Tests used. According to the selected studies for the present review, the neuropsychiatric symptoms addressed were apathy, depression and anxiety. The following instruments were used to measure apathy: Neuropsychiatric Inventory (NPI),^21^ ^,^ ^23^ ^,^ ^26^ ^,^ ^28^ ^,^ ^29^ Apathy Scale,^26^ and the Apathy Evaluation Scale.^24^ For assessing depressive symptoms, five papers used the Geriatric Depression Scale,^23^ ^,^ ^24^ ^,^ ^26^ ^,^ ^27^ ^,^ ^29^ whereas only one study measured depression with the Minimum Data Set Depression Rating Scale.^22^ Two studies used the Beck Depression Scale (GDS)^23^ ^,^ ^29^ and one applied the Cornell Scale for Depression in Dementia (CSDD).^25^ Lastly, the Hamilton Anxiety Rating Scale (HAM-A)^25^ was used to evaluate anxiety.

Only one study used the NPI to measure sleep disturbances and eating disorders, as well as the Mini Nutritional Assessment (MNA) for measuring nutritional status, although no significant differences were found.^21^

With respect to the intervention period of studies, four investigations lasted for 12 weeks,^22^ ^,^ ^25^ ^-^ ^27^ followed by 24 weeks,^21^ ^,^ ^24^ 10 weeks^28^ and lastly, 6 weeks.^23^ ^,^ ^29^

Unfortunately, only four papers conducted follow-up assessments in order to determine the intervention's possible long-term impact on subjects' psychological health and functional abilities.^21^ ^,^ ^22^ ^,^ ^23^ ^,^ ^29^ Although one study comparing Group 1 (CS + Rivastigmine Transdermal Patch) with Group 2 (Rivastigmine Transdermal Patch) interventions was reviewed, it was not the aim of this review to compare these conducts.^21^ However, depressive and cognitive symptoms, as well as functional status and risk of mortality, decreased among the elderly patients with AD when compared with patients who received the Rivastigmine Transdermal Patch only, during the drug therapy intervention.^21^

## DISCUSSION

The purpose of the present review was to verify the evidence with respect to the benefits of cognitive stimulation in neuropsychiatric symptoms among patients diagnosed with AD. Out of nine studies, eight revealed that CS was beneficial for patients with mild-to-moderate AD in terms of improvement of NPS.^21^ ^,^ ^23^ ^-^ ^29^ Depression, apathy and anxiety were the neuropsychiatric symptoms most assessed. 

Eight investigations evaluated depressive symptoms, six of which revealed statistically significant improvement in depression scores after the intervention period.^21^ ^,^ ^23^ ^,^ ^25^ ^,^ ^26^ ^,^ ^29^ This was confirmed by comparison of control and intervention groups, revealing that the intervention group had lower scores of depression, as did their caregivers, who also reported lower burden relative to levels assessed before the interventions,^23^ providing evidence that cognitive stimulation tends to attenuate depressive symptoms.

A study conducted in 2016 reported little difference in depression scores on comparison of both control and intervention groups. However, this finding may be due to the fact that depression level may not have been sufficiently diagnosed in the elderly AD patients to allow an accurate conclusion.^24^ The authors pointed out that the GDS assessment, used to identify depression in the elderly, is not a specific tool for screening depression in patients with dementia.^24^ Moreover, only one paper used a specific validated instrument for measuring apathy^25^ and likewise, only one study used a specific instrument for measuring anxiety in AD patients.^21^ It is important to bear in mind that these assessments should only be used as screening tests.

Three papers assessed the effect of a CS program on apathy in patients with AD. The first, a pilot study conducted in 2012 comprising 14 patients was undertaken to investigate the effectiveness of cognitive stimulation, physical activity, and socialization in AD patients' symptoms. The second, a paper published in 2011, involved 39 patients diagnosed with mild AD and to evaluated the effectiveness of an art therapy technique in AD symptoms. The third, a 2010 study in 32 patients with mild-to-moderate AD assigned to a CST group was carried out to determine the effects of the intervention. All three studies reported statistically significant efficacy in lowering apathy scores after treatment.^25^ ^,^ ^27^ ^,^ ^28^

Two studies assessed anxiety. The first was performed in 2014 with 90 AD patients and evaluated anxiety symptoms, reporting significant positive outcomes at a 6-month follow-up.^21^ Another study in 2015 also observed significant improvement in anxiety after cognitive stimulation.^25^

Overall, significant positive outcomes for depression, apathy and anxiety were reported, yielding strong evidence that cognitive stimulation can be effective in these NPS. Two studies observed that AD patients that participated in physical activity, cognitive stimulation and socialization intervention groups, had lower progression of AD symptoms and showed an improvement in general clinical condition, as did their caregivers.^25^ ^,^ ^26^

These symptoms may often have a gradual characteristic as the illness progresses, therefore CS programs may play an important role in potentially attenuating or stabilizing these symptoms in AD patients. In addition, studies have shown that CS programs also have positive effects on healthy patients and thus may be considered an option for those seeking to prevent the development of dementia and particularly, the development of NPS associated with AD.^5^ ^,^ ^18^

Moreover, a few studies showed that CS could also have benefits in mild cognitive impairment (MCI) or in prodromal phase of the illness, reporting improvements in patients´ psychological well-being.^5^ ^,^ ^18^ ^,^ ^20^ However, it remains unclear whether the effects of a cognitive stimulation program on less prevalent NPS in patients with AD are also positive. One possible reason may be due to the fact that less prevalent symptoms in AD patients are less researched.^7^

Some limitations should be outlined: the investigations reviewed for the present study had relatively small sample sizes.^21^ ^,^ ^22^ ^,^ ^26^ ^-^ ^28^ Two research papers pointed out the heterogeneous composition of the groups, as a potential limitation.^22^ ^,^ ^24^ Another possible limitation was the lack of direct measures for evaluating some NPS,^26^ given that direct measures are believed to provide more accurate information. Lastly, there was significant heterogeneity in the methods used for assessing NPS and this may have been due to the respective paper's publication year, population origin, education level, age, study goals or settings, etc., which may have interfered in a few results. The high variability in evaluation methods can be expected when the main topic is health of the elderly, where the extent and complexity of assessing health problems may have led to the need for specificity in the health assessments of the elderly.^30^ Also, as mentioned above, not all studies used evaluation methods validated for patients diagnosed with dementia, and a more accurate and detailed evaluation method may be necessary for better outcomes. 

The present review provides sound evidence that cognitive stimulation programs represent an effective therapeutic alternative for mild-to-moderate AD patients, revealing positive effects on depression, apathy and anxiety. In summary, CS programs can play an important role in attenuating or delaying progression of symptoms of AD in elderly patients. In addition, these programs can be associated with combined approaches based on different modalities, such as physical activity and social and emotional support, focusing on socialization. The effects tend to promote improvement and enhancement in the quality of life of AD elderly patients, as well as that of their informal and formal caregivers. 
